# Inactivation of *CDK12* Enhances Mitochondrial Efficiency to Suppress DNA Damage

**DOI:** 10.1111/jcmm.71101

**Published:** 2026-03-27

**Authors:** Aishwarya Gondane, Shivani Yalala, Jing Liang, Sonja Saira, Harri M. Itkonen

**Affiliations:** ^1^ Department of Biochemistry and Developmental Biology, Faculty of Medicine University of Helsinki Helsinki Finland; ^2^ Department of Clinical Molecular Biology, EpiGen, Institute of Clinical Medicine University of Oslo Oslo Norway; ^3^ EpiGen, Medical Division Akershus University Hospital Lørenskog Norway

**Keywords:** acquired resistance, cyclin‐dependent kinase 12, genomic instability, metabolism, prostate cancer, SLAM‐seq, transcription elongation

## Abstract

Inactivation of cyclin‐dependent kinase 12 (CDK12) characterizes a subset of prostate cancers but it is not understood how cells adapt to declining activity of this major transcription elongation kinase. To probe this response, we developed a cell line resistant to an inhibitor targeting CDK12 and its paralog, CDK13. CDK13 can compensate for the loss of CDK12, which is why we used the dual inhibitor THZ531. Targeted drug screening of the parental and resistant cell lines revealed cross‐resistance to other transcriptional kinases but no clear acquired point of vulnerability. Using genome‐wide mapping of mRNA‐stabilization based on metabolic labelling of RNA, we report selective mRNA stabilization of factors promoting oxidative phosphorylation in the resistant cells. We go on to show that loss of CDK12 activity enhances ATP production both in cell line models and in patient tumours. Finally, we show that dual inhibition of CDK12/13 results in excessive phosphorylation of the DNA damage H2AX in prostate cancer cells but not in our CDK12/13 inhibitor‐resistant model system. In brief, we propose that inactivation of *CDK12* rewires cellular energy metabolism to suppress DNA damage.

## 1 Background

Prostate cancer is the most common cancer in males [1]. The disease is driven by the nuclear hormone transcription factor, androgen receptor, which is also the primary target to successfully control prostate cancer [2]. Disappointingly, resistance to anti‐androgens occurs frequently, and patients are left with no targeted therapies to control their disease. Better understanding of the anti‐androgen‐resistant disease holds promise to control prostate cancer.

Cancer cells exhibit high levels of transcription, and it is therefore unexpected that one of the transcriptional kinases, cyclin‐dependent kinase 12 (CDK12), is inactivated in prostate cancer. CDK12 sustains phosphorylation of RNA polymerase II (RNA Pol II) to promote transcription elongation of the particularly long genes [3, 4] and its loss confers resistance to anti‐androgens [5]. The other two major transcriptional kinases are CDK7 and CDK9, which regulate transcription initiation and release of the polymerase from promoter‐proximal pausing, respectively [6]. Many of the long genes affected due to CDK12 inhibition encode for factors involved in DNA repair, which has been proposed as causal in benefiting cancer cells that have lost this kinase [4]. However, when *CDK12* is inactivated in a stable manner, as it is in the clinical setting or when the gene is removed using CRISPR in vitro, transcription of most of the long genes is restored [7]. Understanding how the decrease in CDK12 activity benefits prostate cancer cells therefore remains obscure.

The characterizing feature of the *CDK12* mutant tumours is genomic instability, and loss of CDK12 activity is associated with poor prognosis. *CDK12* inactivation is most common in prostate cancer but it is also frequently detected in ovarian cancer [8]. Decrease in CDK12 activity can positively regulate transcription to confer a growth advantage by promoting generation of the ligand‐independent isoform of the androgen receptor transcription factor in prostate cancer cells [5], by stimulating transcription of the short genes, at least in response to CDK12 inhibition [3, 4], and truncating mutations of *CDK12* have also been associated with amplification of the *MYC* oncogene [9]. Clearly, decline in CDK12 activity leads to both stable and transient effects to stimulate transcription and alter the overall transcriptional program.

In this project, we developed a CDK12/13 inhibitor resistant prostate cancer model system to understand how cells adapt to decline in the activity of the major transcription elongation kinases. CDK12 and CDK13 have redundant functions, which is why we selected to target both. Our model system shows cross‐resistance against inhibitors of CDK7 and CDK9, the two kinases important for transcription initiation and release of the polymerase from promoter‐proximal pausing, respectively. Using genome wide mRNA‐stabilization analysis (variation of the SLAM‐seq technology), we show that the CDK12/13 inhibitor‐resistant cells selectively stabilize mRNAs involved in mitochondrial metabolism. Importantly, we show that remodelling of mitochondrial metabolism occurs also when *CDK12* is knocked out in vitro and many of the transcriptional changes are present in prostate cancer patient samples with truncating mutations in *CDK12*. We propose that remodelling of mitochondrial metabolism is necessary to mitigate genomic instability when CDK12/13 activity decreases. Accordingly, CDK12/13 inhibitor‐resistant cells do not activate DNA damage response when challenged with compounds targeting CDK12/13, while parental cells do. In the future, it is important to establish if remodelling of mitochondrial metabolism leads to epigenetic changes that generate permissive conditions to support tumour growth.

## 2 Results

### 2.1 Resistance Against CDK12/13 Inhibition Results in Cross‐Resistance Against Compounds Targeting Other Transcriptional Kinases

**FIGURE 1:**
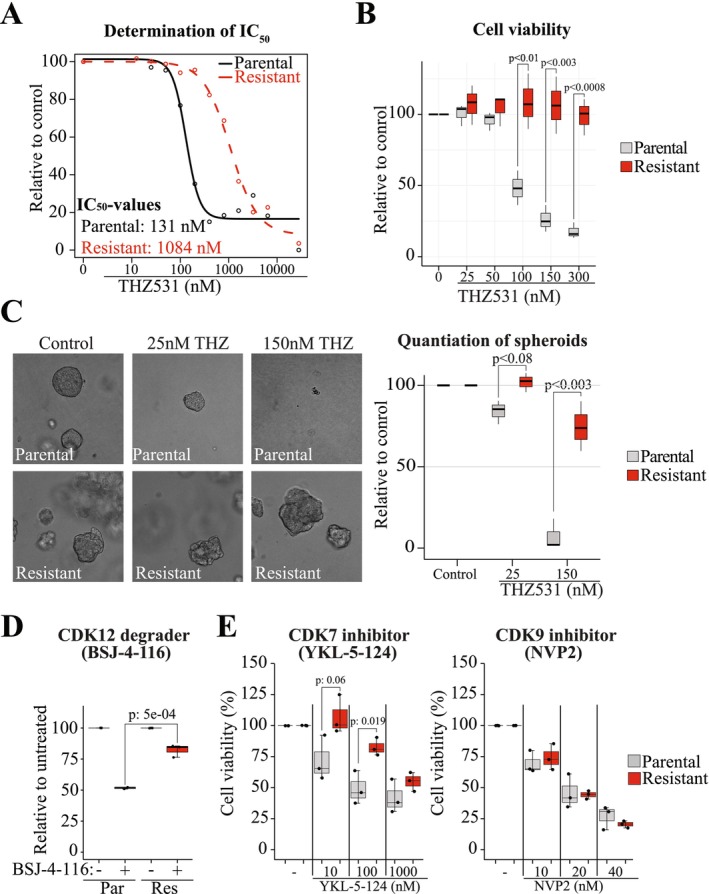
CDK12/13 inhibitor resistant cell line reveals cross‐resistance against inhibitors of other major transcriptional kinases. (A) 22RV1 and CDK12/13 inhibitor resistant cells were treated with increasing dose of THZ531 for 4 days. Cell viability was measured using CellTiter‐Glo assay. IC_50_‐values were determined using dcr package in R [14]. (B) Cell viability after 4 days treatment (3 biological replicates, each with 3 technical replicates, and student's paired samples t‐test was used to assess significance). (C) 22RV1 and CDK12/13 inhibitor‐resistant cells were grown in matrigel to generate spheroids, which were then treated as indicated (3 biological replicates and student's paired samples t‐test was used to assess significance). (D) and (E) Cells were treated with 50 nM BSJ or YKL‐5‐1‐24 or NVP2 for 4 days. Cell viability was measured using CellTiter‐Glo assay. Data presented is from three biological and three technical replicates. Significance was assessed using student's paired samples t‐test.

We set out to understand how prostate cancer cells adapt to declining activity of the transcription elongation kinases CDK12 and CDK13. We reasoned that it is possible to mimic clinical scenario of declining CDK12 activity by treating prostate cancer cells with increasing doses of CDK12/13 inhibitor THZ531 [10]. CDK12 and CDK13 have to some extent redundant functions at least in prostate cancer cells where the loss of *CDK12* causes increased dependency on CDK13 [11]; this is why we wanted to develop a cell line that is resistant to compounds targeting both CDK12 and CDK13. Of note, this cell line would establish how the prostate cancer cells respond to decline in the activity of both of the major transcription elongation kinases, CDK12 and CDK13. We selected the castration‐resistant prostate cancer cell line model 22RV1 for these experiments because this cell line has a specific mutation in the *CDK12* gene known to stimulate androgen receptor (AR) activity [5]. AR is of particular interest in prostate cancer research as it is both a major driver and highly clinically relevant drug target [12, 13]. The missense mutation in 22RV1 cell line and specific patient samples in the *CDK12* gene changing the amino acid from a non‐charged glycine 909 to charged glutamate or arginine stimulates AR activity and thereby confers resistance to anti‐androgens [5]. Initially, when exposed to THZ531, most cells died but after extending the exposure to 6 weeks, we noted that there was no longer apparent cell death. Indeed, the extended exposure to the escalating doses of the CDK12/13 inhibitor led to a 10‐fold increase in the IC_50_‐value against THZ531 (Figure 1A,B). These effects were maintained also in the spheroid culture, where the parental cells failed to grow in the presence of CDK12/13 inhibitor while our resistant model still efficiently formed spheroids (Figure 1C). We note the irregular shape of the spheroids derived from CDK12/13 inhibitor resistant cells when compared to parental cells, which likely indicates significant rewiring of the resistant cells. The acquired resistance is not explained by increased expression of CDK12 itself as there was no robust difference in its expression between the parental and resistant cells (Figure S1). The predominant activity of CDK12 is to phosphorylate RNA Pol II on Ser‐2 [15], and we therefore assessed how the acute and chronic presence of the dual inhibitor of CDK12/13 (THZ531) affects this. Acute treatment with THZ531 led to an almost complete loss of RNA Pol II phosphorylation on Ser‐2, and we also detected a clear decline in this phosphorylation event in our resistant cell line (Figure S1). To further validate these data, we used a structurally unrelated compound to THZ531, the CDK12 degrader BSJ‐4‐116 [16], to confirm a highly significant reduced sensitivity of our novel cell line when compared to the parental cell line (Figure 1D). CDK12 is particularly important for the expression of many DNA damage response factors [15, 17], and we therefore assessed if the expression of one of these (RAD51) is affected in our resistant cell line. As expected, acute CDK12/13 inhibition resulted in a clear decrease in the RAD51 levels, while its expression was restored to basal levels in our resistant cells (Figure S1). Notably, this is similar to the clinical setting, where the expression of most of the long genes is similar between wild type and *CDK12* mutant prostate tumours [18]. In addition, while the acute CDK12/13 inhibition induced a robust increase in phosphorylation of the DNA damage marker H2AX, our resistant cells did not exhibit elevated levels.

We reasoned that the acquired resistance to CDK12/13 inhibition renders cells more dependent on the other major positive regulators of RNA polymerase II (RNA Pol II), CDK7 and CDK9. CDK7 is important during transcription initiation, while CDK9 phosphorylates the polymerase to release it from promoter‐proximal pausing [19]. In contrast to our hypothesis, CDK12/13 inhibitor‐resistant cells were significantly less sensitive to CDK7 inhibition and also modestly less sensitive to compounds targeting CDK9 (Figure 1E). These data show that the resistant cells do not become more dependent on the other two major transcriptional kinases but instead become less sensitive against compounds targeting any of the major transcriptional kinases.

We note that the developed cell line represents a model where cells survive the declining activity of both CDK12 and CDK13. Nevertheless, our novel model system enables us to ask if the resistance to a compound interfering with transcription elongation results in acquired sensitivity and/or cross‐resistance to other compounds using more systematic screening.

### 2.2 Acute but Not Chronic Loss of CDK12/13 Activity Sensitizes Cells to Compounds Interfering With Cell Cycle

**FIGURE 2:**
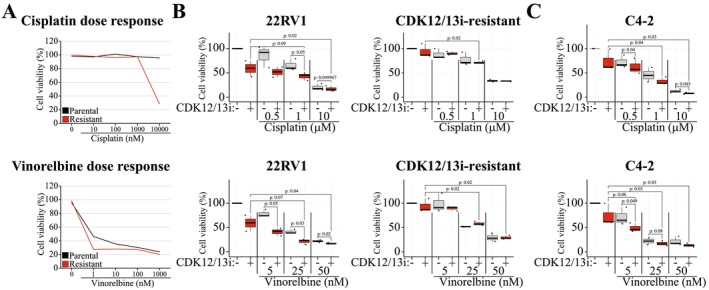
Acute inhibition of CDK12/13 sensitizes prostate cancer cells to compounds interfering with S Phase progression. (A) Results from high‐throughput screen identified Cisplatin and Vinorelbine as compounds with stronger effect on cell line resistant to CDK12/13 dual inhibitor. Cells were treated as indicated for 4 days and viability was measured (the screen was done only once). (B) and (C) Validation of drug screen data. 22RV1, CDK12/13 inhibitor‐resistant and C4‐2 cells were treated with Cisplatin or Vinorelbine with the indicated doses in the presence and absence of CDK12/13 inhibitor. Cell viability was measured after 4 days treatment (3 biological replicates, each with 3 technical replicates, and student's paired samples t‐test was used to assess significance).

We performed a small‐scale targeted drug screen using compounds either already approved or in clinical development to identify potential actionable vulnerabilities associated with resistance to CDK12/13 inhibition (Table S1). The screen was performed on both parental and resistant cells in the absence of the CDK12/13 dual inhibitor THZ531, and the treatment time was 4 days. Interestingly, we discovered that CDK12/13 inhibitor‐resistant cells have a modestly increased sensitivity against several compounds interfering with mitosis (Table S1). However, the increased sensitivity was at best modest, which increases the risk of false positives. For validation, we selected two compounds, Cisplatin and Vinorelbine (Figure 2A). Both of these compounds interfere with S Phase, either by crosslinking DNA (Cisplatin) [20] or by affecting the tubulin assembly (Vinorelbine) [21]. Disappointingly, our validation experiments did not identify any clear acquired sensitivity in CDK12/13 inhibitor resistant cells when compared to parental cells (Figure 2B). Interestingly, however, we do note that co‐treatment of the parental cells with the dual inhibitor of CDK12/13 significantly sensitized them to both Cisplatin and Vinorelbine (Figure 2B). To validate that acute depletion of CDK12/13 activity can sensitize castration‐resistant prostate cancer cells to compounds interfering with S‐Phase, we treated C4‐2 prostate cancer cells with Cisplatin and Vinorelbine. Indeed, inhibition of CDK12/13 activity significantly sensitized also C4‐2 cells to these compounds (Figure 2C). Our small‐scale combinatorial lethality screen proposes that cells experiencing acute loss of CDK12/13 activity have difficulties in completing the S Phase.

To summarize our major findings so far, we have developed a CDK12/13 inhibitor‐resistant castration‐resistant prostate cancer model system that is less sensitive to compounds interfering with transcriptional kinase.

### 2.3 Acquired Resistance to CDK12/13 Inhibition Results in Stabilization of Specific mRNAs

**FIGURE 3:**
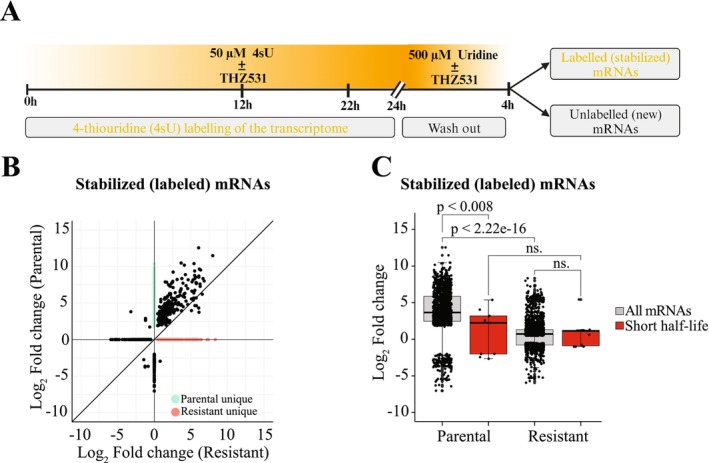
Resistance to CDK12/13 dual inhibition is associated with selective mRNA stabilization. (A) Graphical presentation of the pulse‐chase experiment. Cells were kept in 4‐thiouridine (4sU) for 24 h to assure that the entire transcriptome is labelled. Fresh media along with 4sU was added at 0, 12 and 22 h. After this, 4sU was replaced with uridine in the presence and absence of the CDK12/13 inhibitor THZ531 (150 nM). (B) Correlation of the stabilized mRNAs between the parental and the resistant cell lines in response to CDK12/13 inhibitor treatment (150 nM THZ531). SLAM‐DUNK was used to identify the old mRNA (labelled) and DESeq2 to obtain Log_2_ fold change‐ and significance‐values (*p*‐value < 0.05). Parental unique: Selectively stabilized in parental cell line; Resistant unique: Selectively stabilized in resistant cell line. (C) CDK12/13 inhibitor resistant cells degrade both all and short half‐life mRNAs more rapidly than the parental cells. Short half‐life mRNA data was obtained from Schwanhausser et al. [23]. Short half‐life < 4 h and student's t‐test was used to assess statistical significance.

We hypothesized that the CDK12/13 inhibitor‐resistant cells stabilize the overall transcriptome to tolerate the lowered activity of the major transcription elongation kinases. To probe this, we performed a transcriptome‐wide pulse‐chase labelling experiment relying on the chemistry‐based detection of the mature mRNAs (SLAM‐seq) [22]. We labelled the active transcription with 4‐thiouridine (4sU) for 24 h in the presence or absence of CDK12/13 inhibitor, after which we replaced the label with normal uridine for 4 h and collected the mRNA (Figure 3A, for further details, please refer to the methods‐section). SLAM‐seq is analysed using the SLAM‐DUNK data analysis pipeline, which enables detection of the mRNAs that contain the label and therefore were synthesized in the presence of 4‐thiouridine [24]. In our experimental set‐up, the mRNAs that contain the 4sU label are stabilized.

Unexpectedly, we discovered that the parental cells are more competent in stabilizing their transcriptome in response to CDK12/13 inhibition when compared to CDK12/13 inhibitor‐resistant cells (Figure 3B, upper right quadrant). To further validate this discovery, we compared previously reported genome‐wide mRNA half‐life data [23] to our SLAM‐seq data. Based on the data reported by Schwanhausser et al. [23], we identified the mRNAs that have short half‐lives (less than 4 h, which is also the time point of RNA collection in our SLAM‐seq experiment). As expected, these mRNAs were more rapidly lost than all the significantly affected mRNAs in our parental cells treated with CDK12/13 inhibitor (Figure 3C). In contrast, CDK12/13 inhibitor‐resistant cells had similar mRNA turnover for all of the significantly affected and the short half‐life mRNAs. These data show that the resistance to CDK12/13 inhibition is associated with a more rapid mRNA turnover of most of the transcriptome but a distinct set is also stabilized.

To summarize our major findings so far, we have developed a CDK12/13 inhibitor resistant cell line, and this model system revealed increased mRNA turnover as a feature associated with resistance.

### 2.4 Decrease in the CDK12/13 Activity Rewires Mitochondrial Metabolism

**FIGURE 4:**
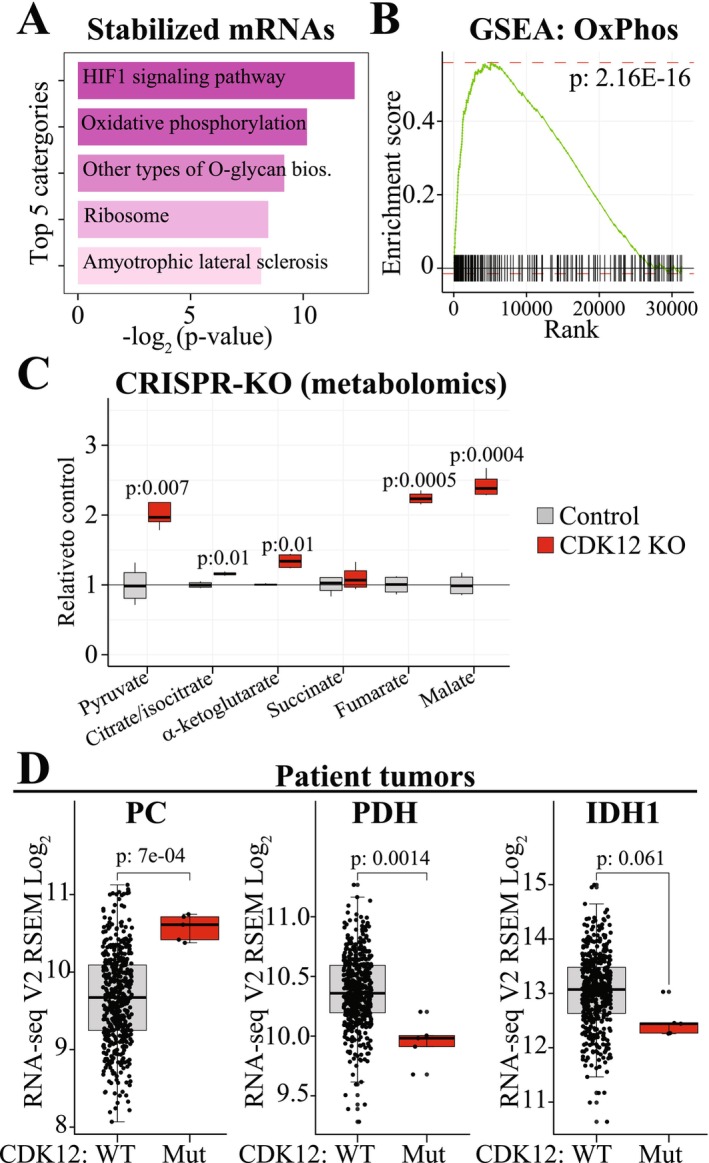
Loss of CDK12 activity rewires mitochondrial metabolism. (A) KEGG pathway enrichment analysis (Enrichr [25]) of the mRNAs selectively stabilized in the CDK12/13 inhibitor‐resistant cells. (B) We analysed our SLAM‐seq data using standard RNA‐seq pipeline to measure the relative abundance of mRNAs between the parental and the CDK12/13 inhibitor‐resistant cells. Oxidative phosphorylation was identified as the most significantly affected gene set in the resistant cells (see also Figure S2). (C) CRISPR‐mediated knockout of CDK12 in C4‐2 castration‐resistant prostate cancer cells increases TCA cycle metabolites. Re‐analysis of the data reported earlier [26]. Student's paired samples t‐test was used to assess the significance. (D) CDK12 inactivation is associated with transcriptional remodelling of the mRNAs belonging to tricarboxylic acid cycle (TCA). Shown are the mRNAs whose expression changes significantly in the *CDK12* mutant prostate cancer samples compared to wild type (see also Table S1). Data was accessed through the cBioPortal and the TCGA Prostate Adenocarcinoma data (TCGA, Firehose legacy) was used. CDK12 WT: No mutation in the *CDK12* gene; CDK12 mut: Truncating mutation in the *CDK12* gene.

We turned our attention to the mRNAs that are selectively stabilized in the cells that tolerate the lowered CDK12/13 activity (Figure 3B, Resistant‐unique marked with red colour). Pathway‐enrichment analysis of these mRNAs identified ‘Oxidative phosphorylation’ as the second most significant KEGG pathway (Figure 4A). To further validate the significance of this discovery, we next identified the most enriched gene sets of the overall transcriptional program using standard RNA‐seq (comparison of the untreated parental cells to the CDK12/13 inhibitor‐resistant cells in the presence of CDK12/13 inhibitor). This approach highlighted ‘Oxidative phosphorylation’ as the most significant gene set in cells with the acquired resistance to the lowered CDK12/13 activity (*p* < 2.16e‐16, Figure 4B and Figure S2). These data show that the tolerance of the lowered CDK12/13 activity is associated with both increased stability and increased overall expression of the genes related to energy metabolism, particularly oxidative phosphorylation.

Oxidative phosphorylation is dependent on the tricarboxylic acid cycle (TCA) for generating reducing equivalents NADH and FADH_2_, which in turn transfer electrons to the mitochondrial respiratory chain [27]. The TCA cycle consists of multiple reactions, and we wanted to establish if a decrease in CDK12 activity stimulates the ability of the cells to perform this cycle as implied by our SLAM‐seq data. To achieve this, we relied on the previously reported metabolomics dataset, where the authors knocked out *CDK12* from castration‐resistant prostate cancer cells and performed metabolite profiling [26]. Interestingly, we noted a highly significant increase in all but one of the TCA metabolites and over a 2‐fold increase in the levels of pyruvate, fumarate, and malate (Figure 4C). These data imply a significant remodelling of the TCA flux. So far, we have relied on cell line data and therefore wanted to probe if our findings are reflected also in the patient samples.

We identified prostate cancer patient tumours with *CDK12*‐inactivation from the TCGA Firehose Legacy dataset to validate the data generated in our novel cell line model. Using the patient data, we evaluated the expression of each of the enzymes required for the TCA cycle in prostate cancer patient samples with either wild type or mutant *CDK12* gene (Table S1). Interestingly, we noted significantly altered expression in three of the TCA cycle mRNAs, pyruvate carboxylase (PC), pyruvate dehydrogenase (PDH), and isocitrate dehydrogenase 1 (IDH1, Figure 4D). The expression of PC was significantly upregulated in the *CDK12* mutant tumours while PDH and IDH1 were downregulated. PC utilizes pyruvate to generate oxoaloacetate, which then depends on PDH‐catalysed acquisition of an acetyl group to synthesize citrate [27]. Finally, IDH1 converts citrate to alpha‐ketoglutarate. The transcriptional profiling data from the *CDK12* mutant tumours map well with the metabolomics data after *CDK12* knockout, where we noted a prominent increase in pyruvate levels but only modest effects for citrate, alpha‐ketoglutarate, and succinate (Figure 4C,D). However, we note that it is not possible to completely recapitulate our in vitro findings in the patient tumours; achieving this would require metabolite profiling of both wild type and *CDK12* mutant tumours, which is difficult to achieve due to the low frequency of *CDK12* mutant tumours. Nevertheless, both our in vitro and publicly available patient data show that altered CDK12 activity is associated with remodelling of TCA cycle gene expression.

### 2.5 Inhibition of CDK12/13 Causes DNA Damage in Parental Cells but Not in CDK12/13 Inhibitor‐Resistant Cells

**FIGURE 5:**
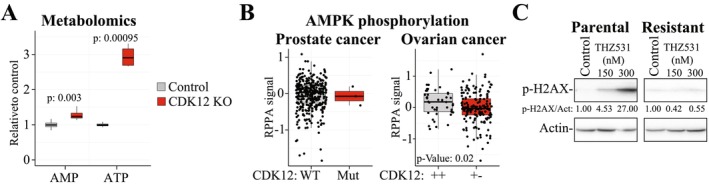
Inactivation of *CDK12* augments metabolic homeostasis. (A) CRISPR‐mediated knockout of *CDK12* in C4‐2 castration‐resistant prostate cancer cells increases ATP levels. Re‐analysis of data reported in [26]. Student's paired samples *t*‐test was used to assess significance. (B) *CDK12* inactivation is associated with lowered phosphorylation of the energy sensor AMPK. Data was accessed through the cBioPortal and the TCGA Prostate Adenocarcinoma (TCGA, Firehose legacy) or Ovarian Serous Cystadenocarcinoma (TCGA, Firehose Legacy) sets were used. CDK12 WT: No mutation in the *CDK12* gene; CDK12 Mut: Truncating mutation in the *CDK12* gene; + +: Both alleles present; + −: One allele lost. We have plotted the reverse‐phase protein array data for AMPK phosphorylation on threonine 172. (C) CDK12/13 inhibitor THZ531 induces phosphorylation of H2AX in the parental cells but not in the CDK12/13 inhibitor‐resistant cells. 22RV1 cells and CDK12/13 inhibitor‐resistant cells were treated with THZ531 for 24 h and protein lysate was collected. Densitometry was used to quantify the signal intensity (*n* = 2).

We sought to acquire further evidence of enhanced mitochondrial activity in cells that have lost the CDK12 activity. We assessed if the loss of CDK12 activity augments cells' ability to maintain high ATP levels using the previously published metabolomics dataset [26]. Indeed, depletion of CDK12 from prostate cancer cells led to a highly significant 3‐fold increase in the ATP levels when compared to the parental cells, while the AMP levels were only modestly increased (Figure 5A). To gain further evidence of favourable energy balance in cells with lowered CDK12 activity, we turned our attention back to the prostate cancer patient tumours and asked if the mutant cells have altered levels of the phosphorylated form of the cellular energy sensor, AMP‐Activated Protein Kinase (AMPK). AMPK is activated by phosphorylation when cells experience an increase in AMP/ATP ratio [28]. Indeed, prostate tumours with truncating mutations in *CDK12* have lower levels of phosphorylated AMPK (non‐significant, Figure 5B), in agreement with our in vitro data. However, this effect is modest, which may in part be explained due to the low number of the *CDK12* mutant cases (only three). We reasoned that this metabolic rewiring should also be present in other cancer types with compromised CDK12 activity. To assess this, we identified ovarian cancer tumours from the Ovarian Serous Cystadenocarcinoma (TCGA, Firehose Legacy) dataset. Indeed, monoallelic loss of the *CDK12* gene, which occurred in 78% of cases, had significantly lower AMPK phosphorylation (Figure 5B). These data further support the notion that decrease in CDK12 activity rewires energy metabolism to maintain high ATP levels.

We hypothesized that decrease in CDK12/13 activity remodels the metabolic activity of cells to sustain genomic integrity. This hypothesis was based on four facts: First, we earlier showed that acute CDK12/13 inhibition sensitizes prostate cancer cells to mitotic poisons (Figure 2). Second, decrease in CDK12 activity alters TCA cycle both in vitro and in patient tumours (Figure 3C,D). Third, *CDK12* inactivation in patient tumours is associated with amplification of *CDK4* and *CCND1* genes [29, 30]. Fourth, depletion of CDK12 activity suppresses transcription of genes involved in DNA repair [3, 4]. If our hypothesis is correct, we would expect parental cells to exhibit excessive phosphorylation of the DNA damage marker H2AX in response to CDK12/13 inhibition, while the CDK12/13 inhibitor‐resistant cells should not. Indeed, treatment of parental cells with CDK12/13 inhibitor THZ531 resulted in a 27‐fold increase in H2AX phosphorylation while no effect was observed in the CDK12/13 inhibitor‐resistant cells (Figure 5C). However, we note that because the chronically treated cells have lost their responsiveness to CDK12/13 inhibition, there may also be other reasons that are at play in this situation.

To summarize the major fundings, here we report a novel prostate cancer cell line model system with acquired resistance to CDK12/13 inhibition, which revealed selective mRNA stabilization as an advantageous adaptation. We note that there is a myriad of mechanisms that may support the acquired resistance; particularly the increased expression of multi‐drug resistance transporters is an obvious one, which also occurs in our model system (as evaluated based on the RNA‐seq data reported in this manuscript). For example, over 5‐fold increased expression of ABCG2 is likely to at least partially explain the cross‐resistance to different compounds (Table S1). Indeed, we confirmed up‐regulation of ABCG2 also at the protein level (Figure S1). Evolution has resulted in development of these transporters to export cytotoxic compounds that have limited structural similarity making their selective inhibition difficult; however, in recent years the advances in understanding the structures of these proteins has enabled development of compounds interfering with their activity [31, 32, 33]. In principle, co‐targeting of a multi‐drug resistance transporter with cancer cell‐selective therapy presents an attractive approach to control the tumour.

Curiously, we discovered an acquired resistance against other major transcriptional kinases and selective stabilization of the transcriptome as features of CDK12/13 inhibitor resistant cells (Figures 1E and 3B). It currently remains unclear how the selective stabilization of the transcriptome occurs. The levels of any mRNA are controlled at multiple levels, including the overall degradation rate of mRNAs, higher basal transcription (of the mRNAs involved in mitochondrial metabolism potentially in this case), and selective stabilization of specific mRNAs. Most likely, more than one mechanism is involved, but we propose that increased transcription of the genes involved in mitochondrial metabolism is particularly important because ‘Oxidative phosphorylation’ was significantly enriched when we compared the overall transcriptomes of parental and CDK12/13 inhibitor‐resistant model systems (Figure 4B). These data propose that transcriptional adaptations are the key features leading to tolerance against the lowered CDK12/13 activity. Indeed, we go on to show that a decrease in CDK12 activity leads to transcriptional remodelling of cellular energy metabolism to augment mitochondrial activity both in vitro and in patient tumours (Figures 4C,D and 5A,B). Earlier, we reported that transcriptional stress rapidly affects mitochondrial activity, which further underscores the intimate link between transcription and metabolism [34]. We note that the mitochondrial metabolism appears to be blunted in prostate cancer patient tumours that harbour truncating mutations in the *CDK12* gene (Figure 4D) and propose that this is to limit the overall flux through the TCA cycle to suppress generation of reactive oxygen species.

In brief, our study is the first to report remodelling of mRNA turnover as an adaptation mechanism to transcriptional stress. The epigenetic status of the *CDK12* mutant tumours should be significantly altered due to remodelling of metabolic flux, which is an interesting topic for future studies. We conclude that *CDK12* inactivation stabilizes selective part of the transcriptome to support energy homeostasis of the affected cell.

## 3 Discussion

Here, we developed a novel prostate cancer model to understand how these cells adapt to decline in the activity of the major transcription elongation kinases CDK12 and CDK13 (Figure 1A–C). Our model highlights the critical differences in the behaviour of prostate cancer cells in response to the acute and chronic presence of the CDK12/13 inhibitor THZ531. Our most striking finding is a mechanism by which the cells can adapt to the decrease in DNA repair activity through selective mRNA stabilization to promote mitochondrial ATP‐generation.

We show that co‐treatment with CDK12/13 inhibitor and compounds targeting cell cycle form combinatorial lethal pairs in CRPC cells (Figure 2), and these data are in agreement with a recent CRISPR screen identifying many regulators of cell cycle as combinatorial lethal targets with *CDK12* knockout in prostate cancer cells [11]. However, when cells have adapted to the presence of CDK12/13 inhibition, the combinatorial anti‐proliferative effects are no longer seen. These results align with clinical findings, which show that inactivation of *CDK12* in prostate cancer patient tumours is associated with amplification of the genes encoding for *CDK4* and *Cyclin D* [29, 30]. Together with the literature, our data propose that specific regulators of the cell cycle may become points of vulnerability in cells that have lower CDK12 activity. It would therefore be of great interest to assess if the clinically utilized CDK4/6 inhibitor Palbociclib is synthetically lethal to *CDK12* mutant tumours. Due to the cross‐resistance against other transcriptional kinases (Figure 1E), we reasoned that the resistant cells stabilize their transcriptome, which should decrease dependency on the basal transcriptional machinery.

Earlier, it has been established that the oncogenic mutations in the *CDK13* gene cause aberrant mRNA stabilization [35]. Here we show that resistance to CDK12/13 inhibition results rather in increased mRNA turnover (Figure 3B). It is possible that CDK12 and CDK13 regulate mRNA stability in an antagonizing manner; however, the earlier study relied predominantly on a non‐mammalian system, and these functions may differ between organisms. Curiously, we noted that the resistant cells stabilize a distinct set of mRNAs (mRNAs marked with red color in Figure 3B).

The transcriptional analysis of our THZ531‐resistant prostate cancer model additionally points towards a further adaptive rewiring highlighting the enrichment of key survival pathways: Hypoxia‐Inducible Factor‐1 alpha (HIF1‐α) signalling and oxidative phosphorylation (Figure 4A). HIF1‐α is a master regulator that drives tumour adaptation to hypoxic and stressful microenvironments, promoting survival, metabolic shift, and, interestingly, HIF1‐α can also induce the expression of drug efflux pumps. Here, we show that the acquired resistance to CDK12/13 inhibition is associated with the upregulation of ABCG2 (Figure S1), which in turn lowers the effective intracellular concentration of the inhibitor. The role of HIF1‐α in *CDK12* mutant tumours should be further explored in the future.

We hypothesize that remodelling of the TCA cycle in the CDK12/13 resistant cells is an adaptive mechanism to suppress production of the reactive oxygen species. This proposal is based on two notions. First, acute decrease in CDK12 activity suppresses the expression of the DNA damage response genes [3, 4]. Second, prostate cancer cells accumulate mutations in the mitochondrial genome with a 55‐times higher rate than in the autosomes [36], and the normal prostate cells have an attenuated mitochondrial flux to promote secretion of citrate [37]: this means that prostate cancer cells may not have fully functional mitochondria. Accordingly, we show that the mRNAs selectively stabilized in THZ531‐resistant cells are significantly associated with oxidative phosphorylation (Figures 3B and 4A,B). Enhanced mitochondrial efficiency is seen also when the *CDK12* gene is depleted using CRISPR (Figures 4C and 5A), and the expression of specific TCA‐cycle enzymes is significantly rewired in the patient tumours with *CDK12* inactivation when compared to the wild type tumours (Figure 4D). We propose that the lowered CDK12 activity causes an evolutionary pressure towards allowing survival of cancer cells that have efficient mitochondria at the expense of those that do not possess fully functional mitochondria.

CDK12 has both tumour suppressor and oncogenic functions but mutations in the gene are generally associated with a more aggressive disease [38, 39, 40, 41]. Here, we show that resistance to THZ531 is associated with decreased MYC‐ and mTORC1‐signalling (Figure S2). Earlier, low dose CDK12 inhibition has been shown to augment MYC activity [42], but excessive over‐expression of MYC can also sensitize cancer cells to CDK12 knockdown [43]. In addition to phosphorylating RNA Pol II, CDK12 also phosphorylates the mRNA 5′ cap‐binding repressor, 4E‐BP1, which in turn enhances translation of the mRNAs dependent on mTORC1 [44]. Curiously, CDK12 thereby affects cells not only by stimulating transcription elongation but also by directly affecting translation; however, currently it remains unclear what regulates sub‐cellular localization of CDK12 to allow these distinct functions and whether RNA Pol II or translation machinery are the preferred substrates. Nevertheless, these data further add to the complexity of CDK12 biology, which clearly can be either tumour suppressive or oncogenic.

In summary, these findings show that THZ531 acts acutely by interfering with nascent transcription and chronically by reshaping mRNA stability, in line with the established functions of CDK12 and CDK13 as regulators of RNA polymerase II elongation. Our novel cell line model together with the earlier data show that prostate cancer cells adapt to a decline in CDK12 activity by altering metabolic flux when DNA repair capacity is, particularly acutely, but also chronically, impaired.

## 4 Materials and Methods

### 4.1 Cell Culture, Proliferation Assays and Western Blotting

C4‐2 (CVCL_4782) and 22RV1 (CVCL_1045) cell lines were obtained from the American Tissue Culture Collection (ATCC), and these cell lines were maintained in RPMI medium supplemented with 10% fetal bovine serum (FBS). Original vial from ATCC was expanded, frozen down, and new vial was thawed frequently to ascertain the identity of the cell line. All the experiments were done in mycoplasma‐free conditions. CDK12/13 inhibitor‐resistant cell line was derived from 22RV1 (CVCL_1045) cells through continuous exposure to increasing doses of THZ531, and the cell line is maintained as the parental one except 150 nM THZ531 is included in the culture media. Briefly, 22RV1 cells were cultured in RPMI+FBS medium supplemented with 75 nM THZ531 for 2 weeks. Later, these cells were cultured in RPMI+FBS medium with 150 nM THZ531 to select cells which confer resistance to the CDK12 inhibitor for 4 weeks. This cell line was generated as part of the project and does not therefore have a ‘Research Resource Identifier’.

For western blot experiments, cells were grown in 6‐well plates for 24 h, after which they were treated as indicated in the figure‐legends. Cell lysates for western blot were prepared as previously described (except no sonication) [45]. Antibodies used are from ABClonal: ABCG2 (A5661), Santa Cruz Biotechnology: RAD51 (sc‐53428), p‐H2AX (sc‐517348), from Abcam: CDK12 (246887), Actin (ab49900), and from Cell Signalling Technology: p‐Ser 2 RNA polymerase II (13499). Compounds were obtained from MedChemExpress.

### 4.2 Spheroid Experiments

Cells were plated in Matrigel (Corning, catalogue number: 354234) and allowed to grow for 1 day before treatment. Spheroids were treated for 7 days as indicated. To visualize the spheroids, we imaged them using the Zeiss AxioObserver inverted widefield microscope (Plan‐Apochromat 20×/0.8). CellTiter‐Glo 2.0 assay (Promega) was used to lyse the organoids, and the luminescence signal was recorded. The data generated is from three biological replicates.

### 4.3 SLAM‐Seq

For metabolic labelling experiment (SLAM‐seq), 22RV1 and CDK12/13 inhibitor‐resistant cells were grown in 6‐well plates for 1 day. The next day, cells were treated with 50 μM 4‐thiouridine (4sU, obtained from ThermoFisher Scientific) with and without 150 nM THZ531 for 24 h. The fresh media along with fresh 150 nM THZ531 and fresh 50 μM 4sU were added at 0, 12 and 22 h. After 24 h of treatment and labelling, the media was replaced with the one containing 500 μM uridine for 4 h with and without 150 nM THZ531. RNA was purified using the Amersham RNAspin Mini Kit (catalogue number 25050071) according to manufacturer's instructions except 0.1 mM DTT was included in all the buffers to protect the thiol‐group. Purified RNA was alkylated as previously described [22]. Finally, library‐preparation and sequencing were purchased as service from Lexogen. In brief, libraries were prepared using QuantSeq 3′ mRNA‐Seq library preparation kit (FWD) for Illumina sequencing and sequencing was performed using SR200 High Output sequencing on Illumina NextSeq 2000.

### 4.4 Bioinformatics

For RNA‐ and SLAM‐seq analysis, the raw fastq files were trimmed using cutadapt and aligned to human genome HG38 using bowtie2 [46], and Samtools was used to convert SAM‐files to BAM‐files [47]. Differential gene expression analysis of standard RNA‐seq was performed using DESeq2 [48]. For analysis of the SLAM‐seq data, we used SLAM‐DUNK [24]. Fgsea [49] was used to perform gene set enrichment analysis (GSEA). Threshold of padj‐Value < 0.05 was selected to define the significantly enriched hallmark pathways. R4.2.0 was used to identify stabilized mRNAs and to generate some of the scatter plots, boxplots, and histograms.

## Author Contributions

A.G. generated and validated the resistant cell line, generated SLAM‐seq data and participated in its analysis. She also contributed to conceptualization of the cell line model and its responses and performed a significant part of the experimental validation. S.Y. performed validation of the drug screen data in 22RV1, CDK12/13 inhibitor‐resistant cells, and C4‐2 cell lines, and validated some of the data using patient data. J.L. prepared both parental and resistant cells for the small‐scale targeted drug screen, analyzed part of the SLAM‐seq data and evaluated the expression of the TCA cycle genes in patient samples using data available via cBioPortal. S.S. characterized acute and chronic response to CDK12/13 inhibition by performing western blot. A.G. obtained resources for the project. H.M.I. conceptualized the project, obtained resources, participated in generating the stable cell line and SLAM‐seq sample collection, supervised experiments and wrote the manuscript. All authors read, commented and approved the manuscript.

## Funding

A.G. is supported in part by The Magnus Ehrnrooth foundation and the Young investigator from Biomedicum. HMI is grateful for the funding from the Academy of Finland (Decision nrs. 331324, 358112 and 335902), the Jenny and Antti Wihuri Foundation, the Cancer Foundation Finland and the Sigrid Juselius Foundation. The funders had no role in the conceptualization, design, data collection, analysis, decision to publish, or preparation of the manuscript.

## Ethics Statement

The authors have nothing to report.

## Consent

The authors have nothing to report.

## Conflicts of Interest

The authors declare no conflicts of interest.

## Supporting information


**Figure S1:** Acute and chronic response to CDK12/13 inhibition. 22RV1 cells were treated with 150 nM THZ531 for 24 h or at least 4 weeks. Data is representative of two biological replicates.
**Figure S2:** Gene set enrichment analysis (GSEA) identifies oxidative phosphorylation as the most significant gene set in the CDK12/13 inhibitor‐resistant cells. GSEA of RNA‐seq data using fgsea. Differentially expressed genes between the parental and the resistant cells were identified and subjected to GSEA (we analysed our SLAM‐seq data using summarizeOverlap RNA‐seq pipeline without the SLAM‐DUNK step).

**Table S1:** This table has five separate sheets and the instructions on how to read the table are on the first sheet.

## Data Availability

This article has two additional files: supplementary table (along with information required to interpret the table) and a pdf‐file, which contains supplementary figures and the associated legends for the supplementary figure and supplementary table. The datasets supporting the conclusions of this article are included within the article and its additional files. SLAM‐seq data has been deposited to GEO‐database with accession number GSE262268.
