# Supplementary material for: Inactivation of CDK12 Enhances Mitochondrial Efficiency to Suppress DNA Damage
Source: J Cell Mol Med. 2026 Mar 27;30(7):e71101. doi: 10.1111/jcmm.71101 (PMC13140989; doi:10.1111/jcmm.71101)
Supplement: Supplementary file 1 — Figure S1: Acute and chronic response to CDK12/13 inhibition. 22RV1 cells were treated with 150 nM THZ531 for 24 h or at least 4 weeks. Data is representative of two biological replicates. Figure S2: Gene set enrichment analysis (GSEA) identifies oxidative phosphorylation as the most significant gene set in the CDK12/13 inhibitor‐resistant cells. GSEA of RNA‐seq data using fgsea. Differentially expressed genes between the parental and the resistant cells were identified and subjected to GSEA (we analysed our SLAM‐seq data using summarizeOverlap RNA‐seq pipeline without the SLAM‐DUNK step). [file JCMM-30-e71101-s002.pdf]

Supplementary figures and legends for supplementary figures and supplementary table 1

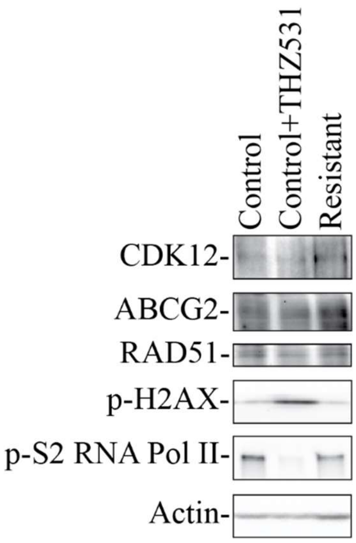

Supplementary figure 1. Acute and chronic response to CDK12/13 inhibition. 22RV1 cells were treated with 150 nM THZ531 for 24 hours or at least four weeks. Data is representative of two biological replicates.

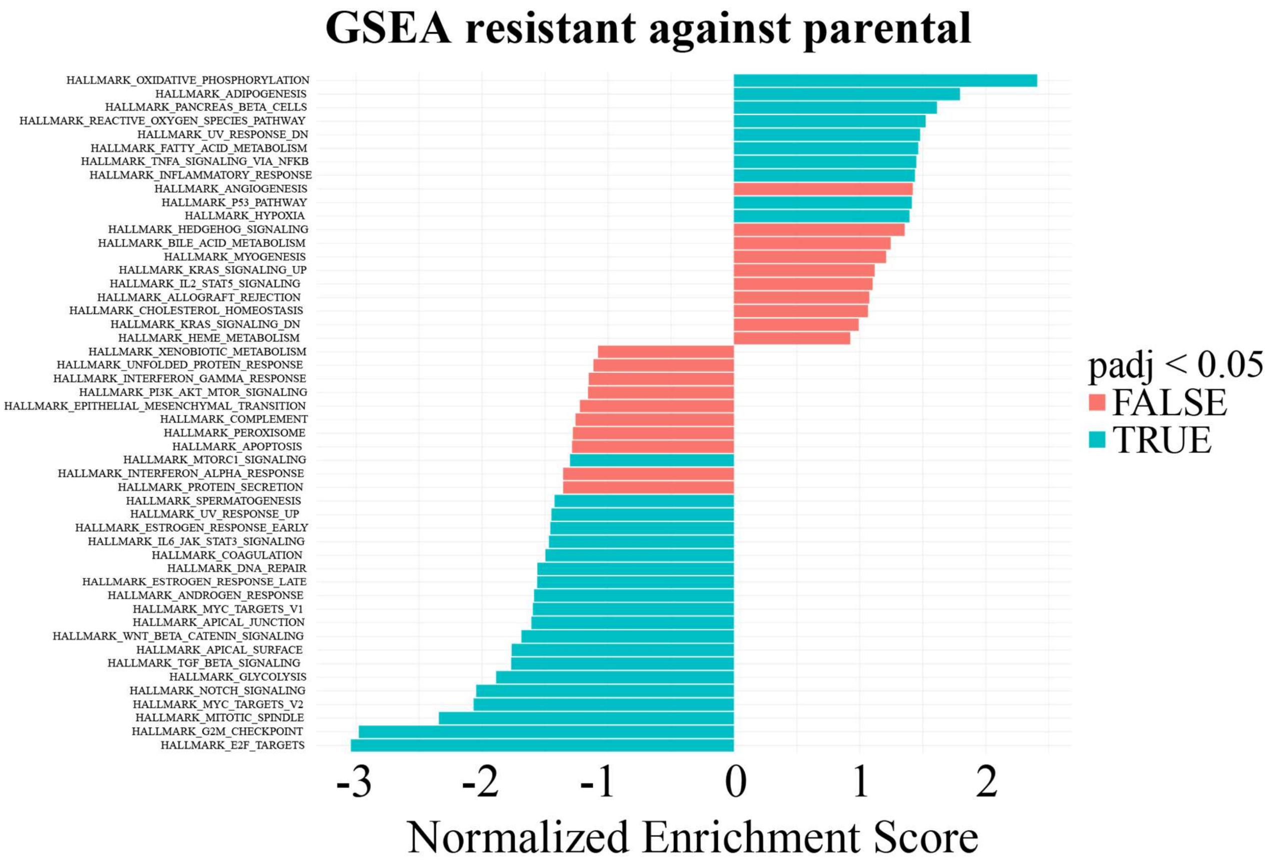

Supplementary figure 2. Gene set enrichment analysis (GSEA) identifies oxidative phosphorylation as the most significant gene set in the CDK12/13 inhibitor-resistant cells. GSEA of RNA-seq data using fgsea. Differentially expressed genes between the parental and the resistant cells were identified and subjected to GSEA (we analyzed our SLAM-seq data using summarizeOverlap RNA-seq pipeline without the SLAM-DUNK step).

Supplementary Table 1. This table has five separate sheets and the instructions on how to read the table are on the first sheet.
